# Inflammatory Parameters in Patients with Suicide Attempts by Drug Overdose: A Comparative Study with a Comparison Group

**DOI:** 10.3390/medicina62020285

**Published:** 2026-01-31

**Authors:** Süleyman Baş, Betül Danapınar, Büşra Çetintulum Aydın, Murat Yeniçeri, Mustafa Can Şenoymak, Kadem Arslan

**Affiliations:** 1 Department of Internal Medicine, University of Health Sciences, Sancaktepe Sehit Prof. Dr. Ilhan Varank Training and Research Hospital, 34785 Istanbul, Turkey; kademarslan@hotmail.com; 2 Department of Internal Medicine, Karabük University Education and Research Hospital, 78200 Karabük, Turkey; betul1danapinar@gmail.com; 3 Department of Internal Medicine, University of Health Sciences, Istanbul Haseki Training and Research Hospital, 34096 Istanbul, Turkey; busracetintulum@hotmail.com; 4 Department of Rheumatology, University of Health Sciences, Kartal Dr. Lutfi Kırdar City Hospital, 34865 Istanbul, Turkey; 4256.gatf@gmail.com; 5 Department of Endocrinology and Metabolism, University of Health Sciences, Sultan 2. Abdulhamid Han Training and Research Hospital, 34668 Istanbul, Turkey; senoymak@gmail.com

**Keywords:** C-reactive protein (CRP), drug overdose, inflammatory parameters, neutrophil-to-lymphocyte ratio (NLR), suicide attempt, systemic immune–inflammation index (SIII)

## Abstract

*Background and Objectives*: The relationship between psychiatric disorders and systemic inflammation remains incompletely understood. Increasing evidence suggests that inflammatory processes may play a role in the biological mechanisms underlying suicidal behavior. This study aimed to investigate the association between classical inflammatory markers and hemogram-derived inflammatory indices in patients who attempted suicide by oral drug overdose. *Materials and Methods*: This retrospective observational comparative study included 343 patients hospitalized following a suicide attempt by oral medication overdose and 421 age- and sex-matched healthy individuals. Serum C-reactive protein (CRP), albumin levels, complete blood count parameters, and derived inflammatory indices, including the CRP-to-albumin ratio (CAR), neutrophil-to-lymphocyte ratio (NLR), systemic immune–inflammation index (SIII), platelet-to-lymphocyte ratio (PLR), and monocyte-to-lymphocyte ratio (MLR), were analyzed. *Results*: Patients with suicide attempts had significantly higher CRP, leukocyte, neutrophil, and monocyte levels compared to the comparison group. CAR, NLR, SIII, and MLR values were also significantly elevated, whereas PLR did not differ between groups. ROC analysis demonstrated that CAR showed the highest discriminative ability for suicide attempt, with high sensitivity and specificity. *Conclusions*: Hemogram-derived inflammatory indices, particularly CAR, were significantly associated with suicide attempts. These easily accessible and low-cost biomarkers may provide additional biological insight into suicide risk assessment. Further prospective studies are needed to confirm these findings.

## 1. Introduction

Relationships between psychiatric diseases and blood tests still remain unclear and are being investigated in current studies. In some psychiatric diseases, such as mood disorders and schizophrenia, it has been proven that some biomarkers change significantly [1,2]. Also, there is substantial evidence to support that changes in the immunomodulatory system play a crucial role in the pathogenic mechanism of mood disorders [3]. It is also known that pathological changes in neuroinflammatory processes are a fundamental pathophysiological mechanism of many chronic diseases, including psychiatric diseases and mental disorders [4].

Suicide deaths are the second leading cause of death in the 15–29 age group. Worldwide, approximately one million people attempt suicide each year, but it is estimated that one in twenty suicide attempts results in a suicide death [5]. Therefore, suicide is a serious global public health problem. Currently, there is no test that allows us to predict suicidality. It may be possible to predict suicidal tendencies in patients with psychiatric disorders with studies to be conducted in this field.

Previous studies have reported that dysregulated neuroinflammatory processes specifically contribute to the pathophysiology of suicidal behavior. For instance, a comprehensive meta-analysis demonstrated that proinflammatory cytokines, particularly IL-6 and IL-1β, are significantly elevated in both the blood and cerebrospinal fluid of suicidal patients, suggesting that inflammation may be an independent risk factor for suicidality [6]. Furthermore, higher levels of C-reactive protein (CRP) have been associated with high-lethality suicide attempts, providing a clinical link between the intensity of systemic inflammation and the severity of the suicidal act [7].

Systemic immune–inflammation index (SIII), neutrophil/lymphocyte ratio (NLR), platelet/lymphocyte ratio (PLR), and monocytes/lymphocyte ratio (MLR) are simple, fast, and inexpensive markers of inflammation that are calculated from hemogram parameters. These parameters have been used as immune-based prognostic scores in different systemic diseases such as malignancy, coronary artery disease, and pancreatitis [8,9,10]. Based on the neuroinflammation hypothesis, the present study aimed to investigate whether these inflammatory parameters differ significantly between patients who have attempted suicide and a comparison group of non-patient peers, thereby evaluating their potential as peripheral markers of suicidality.

## 2. Material and Method

### 2.1. Study Design

This retrospective observational comparative study was conducted at the University of Health Sciences, Sancaktepe Şehit Prof. Dr. İlhan Varank Training and Research Hospital between 1 January 2018 and 30 June 2022. The study included patients who were hospitalized for clinical monitoring following a suicide attempt by oral medication overdose.

A comparison group (non-patient peers) of age- and sex-matched healthy individuals was also included. These individuals were selected from those who presented to the Internal Medicine outpatient clinic of the same institution for routine health examinations or general medical check-ups during the study period and had no history of suicide attempts or psychiatric disorders according to hospital records.

### 2.2. Participants and Selection Criteria

#### 2.2.1. Participants

This study included adult patients aged 18 years and older who were admitted to the Internal Medicine Clinic of the same institution due to a suicide attempt by oral drug overdose and who were hospitalized for clinical monitoring and treatment. Information regarding suicide attempts was obtained retrospectively from the computerized hospital record system and patients’ medical charts. Patients who ingested drugs accidentally, without suicidal intent, or who attempted suicide using methods other than medication were excluded. Individuals with alcohol or psychoactive substance use, acute or chronic inflammatory diseases, infectious, autoimmune or endocrine disorders, and those using medications known to affect bone marrow or immune function were also excluded. Patients with missing hemogram or biochemical laboratory data at admission or discharge were not included in the analysis. Psychiatric diagnoses were recorded based on hospital medical records. Due to the retrospective design of the study and the heterogeneity of psychiatric diagnoses and treatments, subgroup analyses according to specific diagnoses or psychotropic medications were not performed. The comparison group consisted of individuals who presented to the Internal Medicine outpatient clinic of the same institution for routine health examinations or general check-ups. According to hospital records, these individuals had no history of suicide attempts, psychiatric disorders, or chronic systemic diseases. Subjects with acute or chronic inflammatory conditions, infectious diseases, autoimmune or endocrine disorders, or regular medication use were excluded from the study. To minimize potential selection bias, the comparison group was selected from the same hospital setting and were matched for age and sex.

#### 2.2.2. Inclusion Criteria

- To be aged 18 and over;
- To have died by suicide via overdosing;
- To be kept under surveillance by being hospitalized in the Internal Medicine Clinic;
- To have the complete document of the hemogram and biochemical data belonging to the period of application and discharge.

#### 2.2.3. Exclusion Criteria

- Having an overdose without thinking of committing suicide;
- Having an overdose by accident or by mistake;
- The attempted suicide through methods except for medication;
- Having alcohol or psychoactive substance abuse;
- The existence of acute or chronic inflammatory disease;
- The existence of infectious, endocrinologic, or autoimmune disease;
- The use of medicine that affects the function of bone marrow;
- Lack of data about the laboratory data belonging to the period of application or discharge.

#### 2.2.4. Data Collection

Demographic data, including age, sex, and marital status, as well as information on suicide attempt history, type of ingested medications, and documented psychiatric diagnoses were obtained retrospectively from the computerized hospital record system and patients’ medical charts. Psychiatric diagnoses were included in the analysis only when a definitive diagnosis was explicitly documented in the medical records. No additional psychiatric assessments were performed as part of the study due to its retrospective design. Suicide intent was determined retrospectively based on explicit documentation of intentional self-harm in the computerized hospital record system and medical charts, as recorded by the treating physicians at the time of admission. Due to the retrospective nature of the study, the exact time interval between drug ingestion and blood sampling could not be standardized or reliably determined for all patients. Blood samples were obtained at the time of hospital admission as part of routine clinical evaluation.

#### 2.2.5. Medication Classification

Medications used in suicide attempts were categorized into broad, standardized clinical groups based on their primary therapeutic use, including psychiatric medications, analgesics, antibiotics, cardiovascular medications, endocrine/metabolic medications, antidiabetic medications, gastrointestinal system medications, respiratory and allergy medications, and toxic or non-pharmaceutical substances. This classification approach was adopted to improve clinical interpretability and to minimize the use of nonspecific categories by distributing drugs into commonly accepted therapeutic groups, while remaining compatible with the retrospective nature of the study. Detailed pharmacological subclassification was not feasible due to the heterogeneity of ingested agents and the limited availability of standardized information regarding dosage, formulation, and duration of use in routine hospital records. No alternative, herbal, or complementary medicines were identified in the patient files.

### 2.3. The Calculation of the Laboratory Analysis and Inflammatory Indexes

Complete blood count, C-reactive protein (CRP), and albumin levels, which are tested through the venous blood samples belonging to the patients’ application and discharge, are recorded. The inflmatory indexes are calculated by means of the formulas below:

- CRP/Albumin Ratio: (CAR)/Albumin;
- The Neutrophil to Lymphocyte Ratio (NLR): Neutrophil Count/Lymphocyte Count;
- Monocyte Lymphocyte Ratio (MLR): Monocyte Count/Lymphocyte Count;
- Platelet/Lymphocyte Ratio (PLR): Platelet Count/Lymphocyte Count;
- Systemic İmmune-Inflammation Index (SIII): (Neutrophil Count × Platelet Count)/Lymphocyte Count.

These parameters are compared between patient group and the comparison group, and between the period of the application and the discharge of the patient group.

### 2.4. Ethical Approval

This retrospective cross-sectional study is approved by the University of Health Sciences, Sancaktepe Şehit Prof. Dr. İlhan Varank Training and Research Hospital Ethical Committee (Decision No: 2023/03, Date: 16 January 2023).

### 2.5. Statistical Analysis

All the statistical analyses are conducted by using the SPSS 25.0 program (IBM Corp., Armonk, NY, USA). Descriptive statistical methods (mean, standard deviation, median, interquartile range, frequency, percentile, minimum, maximum) in the process of evaluating the data of the study. The normality of the quantitative data was assessed using the Shapiro–Wilk test. The Independent T test is conducted for the normal distribution, and the data that are normally distributed are tested via Mann–Whitney U. The comparison of the categorical variables is analyzed through the Chi-Square test. The Wilcoxon signed rank test is conducted to compare the parameters belonging to the periods of application and discharge of the patient group for paired samples. The performance of the inflammatory parameters in predicting the attempted suicide is assessed through ROC (Receiver Operating Characteristic) analysis and the most appropriate cut-off values by using the Youden index. To account for multiple comparisons in the inflammatory parameter analysis, a Bonferroni correction was applied as a sensitivity analysis; all results remained statistically significant after correction. *p* < 0,05 is accepted as the statistical Significance threshold for all tests.

## 3. Results

A total of 343 patients who attempted suicide by oral drug overdose and 421 healthy individuals were included in the study. The demographic and clinical characteristics of both groups are presented in Table 1. The patient and comparison groups were comparable in terms of age, sex, and marital status (*p* > 0.05). As expected based on the study design, previous suicide attempts and psychiatric diagnoses were present only in the patient group (Table 1).

Among patients, 67.6% had no documented psychiatric diagnosis. The most common diagnoses were mood disorders, followed by anxiety and psychotic disorders (Table 2). Psychiatric medications and analgesics were the most frequently used drug classes in suicide attempts (Table 3).

At admission, patients exhibited significantly higher CRP, leukocyte, neutrophil, and monocyte levels, along with lower albumin levels compared to the comparison group (all *p* < 0.001). No significant differences were observed in lymphocyte or platelet counts. Inflammatory indices including CAR, NLR, SIII, and MLR were significantly elevated in the patient group, whereas PLR did not differ between groups (Table 4).

When admission and discharge values were compared in the patient group, most inflammatory and hematological parameters showed a significant decrease at discharge, indicating a reduction in systemic inflammatory activity (Table 5).

No significant differences in inflammatory parameters were observed between patients with and without a history of previous suicide attempts (Table 6).

**Figure 1:**
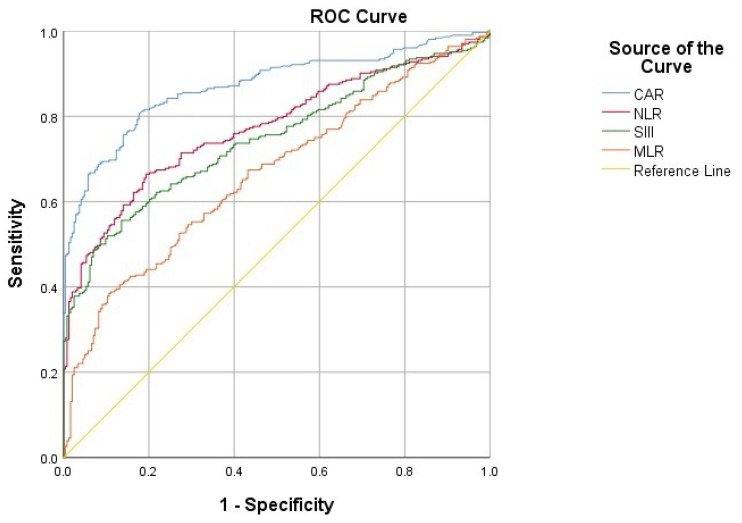
Receiver operating characteristic (ROC) curves of inflammatory markers (CAR, NLR, SIII, MLR) for predicting suicide attempt.

ROC analysis demonstrated that CAR had the highest discriminatory power for suicide attempt (AUC = 0.871), followed by NLR, SIII, and MLR, all of which showed statistically significant predictive performance (Table 7, Figure 1).

## 4. Discussion

This study demonstrates that patients hospitalized after a suicide attempt by oral drug overdose exhibit significantly higher levels of systemic inflammation compared to the comparison group, as reflected by both conventional biochemical markers and hemogram-derived inflammatory indices. In particular, CRP, leukocyte, neutrophil, and monocyte counts, as well as CAR, NLR, SIII, and MLR values, were significantly elevated. These findings support the hypothesis that inflammatory processes are involved in the biological mechanisms underlying suicidal behavior and are consistent with the neuroinflammatory model proposed in psychiatric disorders.

Previous studies have shown increased proinflammatory cytokine activity and immune system activation, particularly in mood disorders and schizophrenia [11,12]. Neuroinflammation-related dysregulation of the hypothalamic–pituitary–adrenal (HPA) axis has been suggested to contribute to impaired stress response, impulsivity, anhedonia, and mood regulation [13]. In this context, several studies have reported elevated peripheral inflammatory markers in individuals with suicidal ideation or attempts, supporting the association between systemic inflammation and suicidal behavior [14,15].

Among the inflammatory markers evaluated, CRP was significantly higher in patients with suicide attempts, in line with previous epidemiological and clinical studies linking elevated CRP levels to suicidal ideation, attempts, and suicide-related mortality [16,17]. As a nonspecific acute-phase reactant influenced by multiple physiological stressors, CRP alone may have limited interpretability in acute clinical settings such as suicide attempts [12]. Therefore, the CRP-to-albumin ratio (CAR), which integrates a positive and a negative acute-phase reactant, may provide a more comprehensive reflection of inflammatory burden [18,19]. In the present study, CAR demonstrated the highest discriminative performance among all indices, likely due to its ability to simultaneously capture CRP elevation and albumin reduction. Previous studies have similarly suggested that CAR is a more stable marker of systemic inflammation and may be associated with depressive symptoms and neuropsychiatric phenotypes [19,20,21].

NLR and SIII were also significantly elevated in patients with suicide attempts, suggesting activation of innate immune responses and relative suppression of adaptive immunity. NLR, reflecting neutrophilia and relative lymphopenia, has been associated with stress-related immune activation and HPA-axis dysregulation [22]. SIII, which incorporates platelet counts into the inflammatory profile, may further reflect thrombo-inflammatory processes accompanying acute systemic stress. Consistent with prior studies, elevated NLR has been reported in major depressive disorder, bipolar disorder, and suicidal behavior [23,24]. However, in the present study, both NLR and SIII showed lower discriminative performance compared to CAR, indicating that these indices may be better interpreted as complementary markers rather than independent predictors of suicidal behavior.

Similarly, MLR was significantly higher in patients with suicide attempts, suggesting a potential role of monocyte-mediated immune activation. Monocytes contribute to peripheral inflammation and may influence neuroinflammatory processes through cytokine release and blood–brain barrier modulation [25,26]. Although previous studies have linked elevated MLR to mood disorders and suicidal behavior [22,27,28], its relatively lower discriminative performance in ROC analysis supports its interpretation as a supplementary indicator within a broader inflammatory profile rather than a standalone biomarker.

In contrast, PLR did not differ significantly between patients and the comparison group. This finding aligns with existing literature reporting inconsistent and weak associations between PLR and psychiatric conditions [29,30,31]. While platelet function has been implicated in suicidal behavior through serotonergic mechanisms [32,33,34], platelet count–based indices such as PLR may inadequately capture these complex neurobiological pathways. Therefore, PLR may have limited utility in psychiatric populations compared to indices integrating broader inflammatory components.

A notable strength of this study is the comparison of inflammatory parameters at admission and discharge, which revealed a significant decline in most markers over time. This pattern suggests that acute stress and toxic exposure associated with suicide attempts may trigger transient inflammatory activation, supporting the concept that inflammation may act as both a state- and trait-related marker in suicidal behavior [17]. The absence of significant differences between patients with and without prior suicide attempts further supports the association between inflammation and acute suicidal behavior rather than long-term vulnerability, although limited statistical power may have influenced this finding.

Overall, these findings highlight the relevance of systemic inflammation in suicidal behavior while emphasizing that hemogram-derived inflammatory indices should be interpreted cautiously. Rather than serving as diagnostic or predictive tools, these markers may provide complementary biological information alongside clinical assessment. Future prospective and multicenter studies with standardized psychiatric evaluations and controlled confounder assessment are required to clarify their clinical utility.

This study has several limitations that should be considered when interpreting the results. First, the retrospective, single-center design restricts the ability to establish causal relationships and may limit the generalizability of the findings. Second, although hemogram-derived indices provide valuable systemic information, more specific inflammatory markers, such as proinflammatory cytokines, could not be evaluated. Furthermore, we were unable to perform multivariable adjustments for potential confounders—including body mass index (BMI), smoking status, socioeconomic factors, and subclinical infections—as these variables were not consistently recorded in routine hospital documentation. Regarding clinical characteristics, the heterogeneity of psychiatric diagnoses and the diverse types of medications ingested precluded a detailed, diagnosis-specific analysis of inflammatory responses. Additionally, suicidal intent was assessed retrospectively based on clinical records rather than standardized psychiatric instruments, which may have introduced misclassification bias. The lack of standardized timing between drug ingestion and blood sampling is another factor that could have influenced the measured inflammatory parameters. Due to these constraints, stratified analyses based on sex or specific drug types were not feasible because of limited statistical power and the risk of multiple-comparison bias. In this context, the ROC-derived parameters, such as AUC, sensitivity, and specificity, should be regarded as exploratory statistical findings rather than definitive indicators of clinical predictive performance. Overall, the findings of this study should be considered hypothesis-generating and exploratory rather than confirmatory or directly applicable for clinical decision-making.

The study also has several notable strengths. With a large sample size and a comparative design, this study is one of the few that comprehensively evaluates systemic inflammation in patients with suicide attempts using both classical biochemical markers and hemogram-derived inflammatory indices. The exclusion of comorbid systemic diseases and the comparison between admission and discharge periods allowed for the assessment of inflammation dynamics specific to the acute phase. Notably, the relatively high discriminative performance of CAR observed in the ROC analysis supports its potential role as an accessible and complementary inflammatory marker within an exploratory research framework, rather than as a standalone tool for clinical decision-making.

## 5. Conclusions

In this study, inflammatory indices such as NLR, SIII, MLR, and especially CAR were found to be significantly higher in patients who attempted suicide by oral drug ingestion compared to the comparison group, with CAR demonstrating the highest discriminative power. These findings support the hypothesis that systemic inflammation may be associated with the biological components of suicidal behavior. Importantly, these inflammatory indices should not be considered diagnostic markers on their own but rather complementary biomarkers that may support clinical assessment in identifying individuals at increased suicide risk. Given their accessibility and low cost, such parameters may offer additional value in clinical settings. Nevertheless, prospective and multicenter studies are required to confirm their clinical utility and validity.

## Figures and Tables

**Table 1:** Demographic and clinical characteristics of patients with suicide attempt and the comparison group.

Characteristics	Patient Group (n = 343)	Comparison Group (n = 421)	*p*-Value
Age (year)	31.12 ± 11.30 ^a^	32.64 ± 13.29 ^a^	0.088 ^b^
Sex (Male/Female)	104 (30.3%)/239 (69.7%)	130 (30.9%)/291 (69.1%)	0.868 ^c^
Marital Status (Married)	176 (51.3%)	214 (50.8%)	0.895 ^c^
Experience of Suicide Attempts (%)	23 (6.7%)	0 (0%)	<0.001 ^c^
Psychiatric diagnosis (%)	111 (32.4%)	0 (0%)	<0.001 ^c^

^a^: Mean ± Standard Deviation, ^b^: Independent T Test, ^c^: Chi-Square Test.

**Table 2:** Distribution of psychiatric diagnoses in patients with suicide attempt.

	Patients (n = 343)
Psychiatric patient story (Yes/No)	
Yes	111 (32.4%)
No	232 (67.6%)
The group of Psychiatric illness diagnosis	
Mood disorders	52 (15.2%)
Anxiety disorders	22 (6.4%)
Psychotic disorders	9 (2.6%)
Addiction disorders	8 (2.3%)
Neurodevelopmental Disorders	6 (1.7%)
Bipolar and related disorders	4 (1.2%)
Obsessive–Compulsive and related disorders	4 (1.2%)
Others/symptom-based diagnoses	4 (1.2%)
Impulse control disorder	2 (0.6%)

**Table 3:** Types of medications used in suicide attempts.

Type of Medication	Patient Group (n = 343)
Psychiatric medication	121 (35.3%)
Analgesics	112 (32.7%)
Antibiotics	41 (12.0%)
Toxic/non-pharmaceutical substances	16 (4.7%)
Endocrine/metabolic medication	13 (3.8%)
Cardiovascular medication	12 (3.5%)
Antidiabetic medication	8 (2.3%)
Gastrointestinal system medication	6 (1.7%)
Respiratory & allergy medication	5 (1.5%)
Others	9 (2.6%)

**Table 4:** Comparison of hematological and inflammatory parameters at admission between patients with suicide attempt group and the comparison group.

Parameter	Patient Group (Admission) (n = 343)	Comparison Group (n = 421)	*p*
CRP (mg/L)	1.60 [0.60–3.33] ^a^	0.24 [0.12–0.60] ^a^	<0.001 ^b,^*
Albumin (g/dL)	4.43 [4.24–4.64] ^a^	4.65 [4.50–4.86] ^a^	<0.001 ^b,^*
Leukocyte (10^3^/µL)	9.39 [7.68–11.07] ^a^	6.94 [6.06–8.10] ^a^	<0.001 ^b,^*
Neutrophil (10^3^/µL)	6.12 [4.78–7.58] ^a^	3.98 [3.30–4.75] ^a^	<0.001 ^b,^*
Lymphocyte (10^3^/µL)	2.33 [1.74–2.93] ^a^	2.34 [2.00–2.73] ^a^	0.677 ^b^
Monocytes (10^3^/µL)	0.52 [0.41–0.64] ^a^	0.42 [0.35–0.51] ^a^	<0.001 ^b,^*
Platelet (10^3^/µL)	263 [224–310] ^a^	266 [233–298] ^a^	0.890 ^b^
CAR	0.37 [0.14–0.80] ^a^	0.04 [0.02–0.12] ^a^	<0.001 ^b,^*
NLR	2.52 [1.84–3.59] ^a^	1.72 [1.34–2.05] ^a^	<0.001 ^b,^*
SIII	685.24 [470–974] ^a^	447.88 [338–575] ^a^	<0.001 ^b,^*
PLR	113.56 [87.07–145.74] ^a^	113.02 [95.66–133.45] ^a^	0.660 ^b^
MLR	0.21 [0.17–0.27] ^a^	0.17 [0.15–0.21] ^a^	<0.001 ^b,^*

^a^: Median [Interquartile Range], ^b^: Mann–Whitney U Test. *: Statistically significant (*p* < 0.05); results also remained statistically significant after Bonferroni correction for multiple comparisons (adjusted *p* < 0.0042). CRP: C-Reactive Protein, CAR: CRP Albumin Ratio, NLR: Neutrophil–Lymphocyte Ratio, SIII: Systemic Immune–Inflammation Index, PLR: Platelet–Lymphocyte Ratio, MLR: Monocyte–Lymphocyte Ratio.

**Table 5:** Hematological and inflammatory parameters at admission and discharge in patients with suicide attempt.

Parameter	Application	Discharge	*p*
CRP (mg/L)	1.60 [0.60–3.33] ^a^	1.00 [0.60–2.17] ^a^	<0.001 ^b,^*
Albumin (g/dL)	4.43 [4.24–4.64] ^a^	4.10 [3.90–4.37] ^a^	<0.001 ^b,^*
Leukocyte (10^3^/µL)	9.39 [7.68–11.07] ^a^	7.96 [6.57–9.41] ^a^	<0.001 ^b,^*
Neutrophil (10^3^/µL)	6.12 [4.78–7.58] ^a^	4.54 [3.74–5.85] ^a^	<0.001 ^b,^*
Lymphocyte (10^3^/µL)	2.33 [1.74–2.93] ^a^	2.38 [1.93–3.02] ^a^	0.061 ^b^
Monocytes (10^3^/µL)	0.52 [0.41–0.64] ^a^	0.51 [0.41–0.61] ^a^	0.350 ^b^
Platelet (10^3^/µL)	263 [224–310] ^a^	241 [200–282] ^a^	<0.001 ^b,^*
CAR	0.37 [0.14–0.80] ^a^	0.24 [0.13–0.53] ^a^	<0.001 ^b,^*
NLR	2.52 [1.84–3.59] ^a^	1.92 [1.38–2.54] ^a^	<0.001 ^b,^*
SIII	685.24 [470–974] ^a^	463.04 [326–647] ^a^	<0.001 ^b,^*
PLR	113.56 [87.07–145.74] ^a^	101.87 [79.13–126.10] ^a^	<0.001 ^b,^*
MLR	0.21 [0.17–0.27] ^a^	0.20 [0.16–0.26] ^a^	0.002 ^b,^*

^a^: Median [Interquartile Range], ^b^: Wilcoxon Signed Rank Test. *: Statistically significant (*p* < 0.05); results also remained statistically significant after Bonferroni correction for multiple comparisons (adjusted *p* < 0.0042). CRP: C-Reactive Protein, CAR: CRP Albumin Ratio, NLR: Neutrophil–Lymphocyte Ratio, SIII: Systemic Immune–Inflammation Index, PLR: Platelet–Lymphocyte Ratio, MLR: Monocyte–Lymphocyte Ratio.

**Table 6:** Comparison of inflammatory parameters in patients with and without previous suicide attempts.

Parameter	Experience of Suicide Attempt (+) (n = 23)	Experience of Suicide Attempt (−) (n = 320)	*p*
CRP (mg/L)	1.55 [0.37–3.20] ^a^	1.60 [0.60–3.35] ^b^	0.364 ^b^
CAR	0.36 [0.08–0.72] ^b^	0.38 [0.14–0.81] ^b^	0.370 ^b^
NLR	2.41 [1.83–3.00] ^b^	2.52 [1.83–3.59] ^b^	0.469 ^b^
SIII	650.79 [435.38–928.20] ^b^	686.70 [471.19–997.83] ^b^	0.812 ^b^
PLR	122.37 [92.34–163.02] ^b^	113.15 [86.84–144.52] ^b^	0.361 ^b^
MLR	0.19 [0.16–0.27] ^b^	0.21 [0.17–0.27] ^b^	0.590 ^b^

^a^: Median [Interquartile Range], ^b^: Mann–Whitney U Test. CRP: C-Reactive Protein, CAR: CRP Albumin Ratio, NLR: Neutrophil–Lymphocyte Ratio, SIII: Systemic Immune–Inflammation Index, PLR: Platelet–Lymphocyte Ratio, MLR: Monocyte–Lymphocyte Ratio.

**Table 7:** Predictive performance of inflammatory parameters for suicide attempt through roc analysis.

Parameter	AUC (95% CI)	Cut-Off	Sensitivity (%)	Specificity (%)	*p*
CAR	0.871 (0.841–0.901)	≥0.13	80.6	82.3	<0.001 *
NLR	0.773 (0.734–0.812)	≥2.14	66.4	80.7	<0.001 *
SIII	0.746 (0.706–0.787)	≥647.16	55.6	86.4	<0.001 *
MLR	0.665 (0.620–0.710)	≥0.23	38.5	89.3	<0.001 *

*: Statistically significant at *p* < 0.05. AUC: Area Under the Receiver Operating Characteristic Curve; CI: Confidence Interval. CAR: C-reactive protein/albumin ratio; NLR: Neutrophil-to-lymphocyte ratio; SIII: Systemic immune–inflammation index; MLR: Monocyte-to-lymphocyte ratio.

## Data Availability

The data presented in this study are available on request from the corresponding author.
